# Plasma dipeptidyl-peptidase-4 activity is associated with left ventricular systolic function in patients with ST-segment elevation myocardial infarction

**DOI:** 10.1038/s41598-017-06514-3

**Published:** 2017-07-21

**Authors:** Jing Wei Li, Yun Dai Chen, Yu Qi Liu, Jin Da Wang, Wei Ren Chen, Ying Qian Zhang, Qiang Ma

**Affiliations:** 1 Department of Cardiology, PLA General Hospital, Beijing, China; 2 Department of Cardiology, Xinqiao Hospital, Third Military Medical University, Chongqing, China

## Abstract

Plasma dipeptidyl-peptidase-4 activity (DPP4a) is inversely associated with left ventricular function in patients with heart failure (HF) or diabetes. However, the association between DPP4a and left ventricular function in ST-segment elevation myocardial infarction (STEMI) patients has not been reported. We studied this association in 584 consecutive STEMI patients at a tertiary referral center from July 2014 to October 2015. DPP4a and plasma N-terminal prohormone of B-type natriuretic peptide (NT-proBNP) levels were quantified by enzymatic assays. The median serum NT-proBNP levels were highest in patients of the lowest tertile (T1) of DPP4a compared with that of the highest tertile (T3) (*p* = 0.028). The STEMI patients in T1 exhibited lower left ventricular systolic function (T1 vs. T3: left ventricular ejection fraction (LVEF): 50.13 ± 9.12 vs. 52.85 ± 6.82%, *p* = 0.001). Multivariate logistic-regression analyses (adjusted for confounding variables) showed that a 1 U/L increase in DPP4a was associated with a decreased incidence of left ventricular systolic dysfunction (LVSD) (adjusted odds ratio: 0.90; 95% CI: 0.87–0.94; *p* < 0.01). In conclusion, low DPP4a is independently associated with LVSD in STEMI patients, which suggests that DPP4 may be involved in the mechanisms of LVSD in STEMI patients.

## Introduction

Left ventricular systolic dysfunction (LVSD), whose representative indicator is left ventricular ejection fraction (LVEF), is an important marker of poorer prognosis in patients after an acute myocardial infarction (AMI), for both in-hospital and long-term follow-up^1, 2^. Plasma biomarkers may provide insight into the pathogenesis of LVSD while also providing prognostic information in MI patients.

Dipeptidyl-peptidase-4 (DPP4) is a widely expressed enzyme, cleaving off proteins containing a position 2 alanine or proline, and thus inactivating peptides^3^. DPP4 plays an important role in glucose metabolism and is responsible for the degradation of glucagon-like peptide-1 (GLP-1) and glucose- dependent insulinotropic peptide. DPP4 regulates immune responses via cleavage of many cytokines and chemokines, such as stromal-cell-derived factor-1 (SDF-1)^4^. Inhibition of DPP4 enhances endothelial regeneration and reduces atherosclerotic progression via the SDF1/chemokine receptor (CXCR)-4 axis^5^. Soluble DPP4 circulating in the plasma exerts catalytic activity cleaving oxyntomodulin^6^ and SDF-1^7^, as does membrane-type DPP4. Soluble DPP4 plays a role in the development of insulin resistance, smooth muscle cell (SMC) proliferation, inflammation, and vasorelaxation^8^. Increased soluble DPP4 levels are associated with coronary artery disease^9^ and heart failure (HF)^10^.

We found that plasma DPP4 activity (DPP4a) is decreased in patients with MI compared with those with only chest pain or unstable angina pectoris. Also, we found that elevated DPP4a is associated with no-reflow phenomenon and a decreased rate of major bleeding events in ST-segment elevation myocardial infarction (STEMI) patients treated with percutaneous coronary interventions (PCI)^11^. However, the relationship between DPP4a and left ventricular function in patients with STEMI has not been reported. The present study was designed to evaluate the relationship between DPP4a levels on admission in STEMI patients treated with PCI, and their left ventricular (LV) function, as assessed by echocardiography during the first 3 days of hospitalization.

## Results

**Figure 1:**
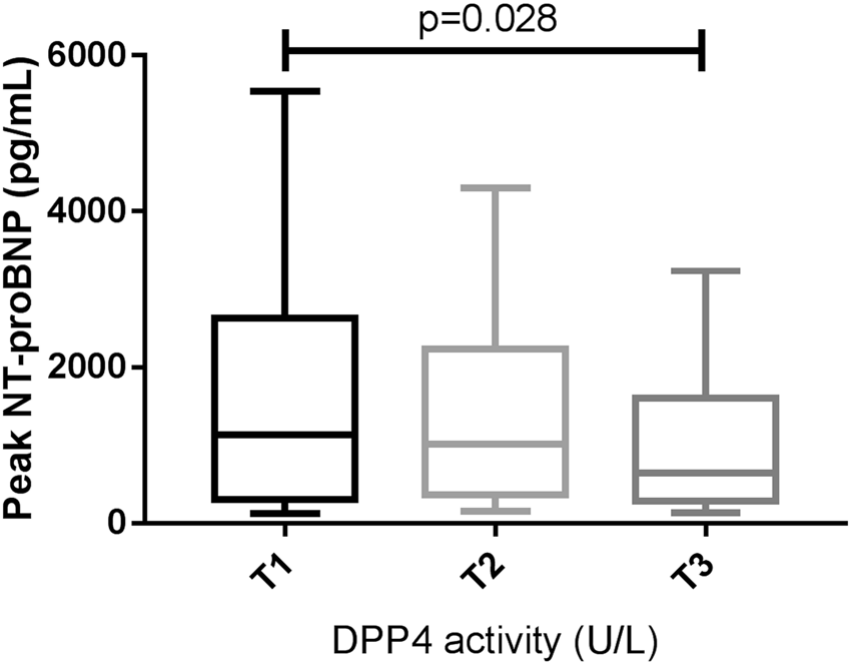
Peak NT-proBNP according to plasma DPP4 activity tertiles in STEMI patients. Values represent as median with interquartile range. Box plots show the 10th and 90th (vertical lines), 25th and 75th (boxes) and 50th (horizontal line).

The demographic, clinical, and echocardiographic parameters of patients with STEMI are presented in Table 1. We divided the patients into three categories according to DPP4a tertiles. Compared to patients in the lowest tertile, patients in the highest tertile were younger, more likely to be current smokers, had higher total low-density lipoprotein (LDL) and high-density lipoprotein (HDL) cholesterol levels, and had lower peak NT-proBNP: 640 (282 to 1607) vs. 1136 (302 to 2629) pg/mL, *p* = 0.02 (Fig. 1). No significant difference of DPP4a was observed between male and female patients (data not shown).

**Table 1:** Demographic, clinical and echocardiographic parameters in STEMI patients according to tertiles of DPP4a.

U/L	Total	T1	T2	T3	*p* value
		<24.00	24.00–31.50	>31.50	
*n*	584	194	196	194	
Age (years)	58.00 ± 11.56	59.02 ± 12.07	58.82 ± 11.55	56.15 ± 10.88	0.03
Male, *n* (%)	491 (84.1)	163 (84.0)	164 (83.7)	164 (84.5)	0.97
BMI	25.80 ± 3.38	25.71 ± 3.31	25.43 ± 3.34	26.27 ± 3.46	0.04
Hypertension, *n* (%)	323 (55.3)	124 (63.9)	97 (49.5)	102 (52.6)	0.01
Hyperlipidemia, *n* (%)	62 (10.6)	24 (12.4)	20 (10.2)	18 (9.3)	0.60
Diabetes mellitus, *n* (%)	118 (20.2)	49 (25.3)	37 (18.9)	32 (16.5)	0.08
Current smoker, *n* (%)	258 (44.2)	72 (37.1)	90 (45.9)	96 (49.5)	0.04
Ex-smoker, *n* (%)	80 (13.7)	35 (18.0)	26 (13.3)	19 (9.8)	0.06
Previous MI, *n* (%)	88 (15.1)	22 (11.3)	29 (14.8)	37 (19.1)	0.10
Previous PCI, *n* (%)	139 (23.8)	52 (26.8)	44 (22.4)	43 (22.2)	0.49
Previous CABG, *n* (%)	5 (0.9)	4 (2.1)	0 (0)	1 (0.5)	0.07
**Medications**, ***n*** (**%**)					
Aspirin	581 (99.5)	194 (100)	194 (99.0)	193 (99.5)	0.37
ACEI/ARB	540 (92.5)	181 (93.3)	179 (91.3)	180 (92.8)	0.75
β-blocker	514 (88.0)	176 (90.7)	170 (86.7)	168 (86.6)	0.36
Clopidogrel	571 (97.8)	191 (98.5)	191 (97.4)	189 (97.4)	0.74
Statin	580 (99.3)	193 (99.5)	195 (99.5)	192 (99.0)	0.77
Nitrate	528 (90.4)	181 (93.3)	170 (86.7)	177 (91.2)	0.08
Diuretic	244 (41.8)	85 (43.8)	86 (43.9)	73 (37.6)	0.36
Total cholesterol (mmol/L)	4.09 ± 1.06	3.91 ± 0.98	4.12 ± 1.13	4.24 ± 1.04	0.01
Triglyceride (mmol/L)	1.56 ± 0.83	1.54 ± 0.80	1.47 ± 0.76	1.65 ± 0.91	0.11
LDL cholesterol (mmol/L)	2.52 ± 0.89	2.38 ± 0.81	2.55 ± 0.97	2.62 ± 0.86	0.03
HDL cholesterol (mmol/L)	1.03 ± 0.28	0.99 ± 0.27	1.02 ± 0.27	1.07 ± 0.29	0.02
Plasma glucose (mmol/L)	6.91 ± 2.65	7.11 ± 2.77	6.90 ± 2.87	6.72 ± 2.26	0.34
CK-MB (ng/mL)	2.71 (1.59–18.78)	2.50 (1.51–10.60)	2.64 (1.61–49.06)	3.02 (1.74–30.07)	0.15
cTNT (ng/mL)	0.18 (0.02–1.62)	0.23 (0.02–1.04)	0.17 (0.02–2.06)	0.19 (0.02–1.94)	0.81
peak NT-proBNP (pg/mL)	931 (329–2143)	1136 (302–2629)	1017 (364–2233)	640 (283–1607)	0.04
Creatinine (umol/L)	78.50 (68.73–90.00)	79.30 (65.00–94.73)	79.55 (71.05–91.83)	76.95 (68.90–85.90)	0.11
**Echocardiographic parameters**					
LVEDVi (mL/m^2^)	57.51 ± 14.82	59.39 ± 17.17	58.10 ± 14.79	55.09 ± 11.80	0.01
LVESVi (mL/m^2^)	28.18 ± 10.75	30.03 ± 13.04	28.40 ± 10.71	26.12 ± 7.49	<0.01
LVMI (g/m^2^)	117.84 ± 26.66	121.41 ± 29.95	118.26 ± 25.79	113.90 ± 23.48	0.02
RWT (%)	0.44 ± 0.06	0.44 ± 0.07	0.43 ± 0.06	0.44 ± 0.05	0.25
SV (mL)	53.07 ± 13.52	52.82 ± 14.08	53.44 ± 13.87	52.94 ± 12.64	0.89
E/A ratio	0.81 (0.68–1.14)	0.81 (0.68–1.14)	0.83 (0.68–1.12)	0.79 (0.67–1.16)	0.08
LVEF (%)	51.47 ± 7.91	50.13 ± 9.12	51.44 ± 7.42	52.85 ± 6.82	<0.01
FS (%)	27.96 ± 4.66	27.37 ± 5.18	27.85 ± 4.44	28.68 ± 4.25	0.02
LVSWi (g/cm^−2^)	0.95 ± 0.22	0.93 ± 0.23	0.95 ± 0.22	0.97 ± 0.20	0.24
LVSD, *n* (%)	60 (10.3)	32 (16.5)	21 (10.7)	7 (3.6)	<0.01

A, maximum late transmitral velocity in diastole; ACEI, angiotensin-converting enzyme inhibitor; ARB, adrenergic receptor binder; BMI, body mass index; BNP, brain natriuretic peptide; CABG, coronary artery bypass grafting; CK-MB, MB isoenzyme of creatine kinase; cTNT, cardiac troponin T; E, maximum early transmitral velocity in diastole; FS, subendocardial fractional shortening; HDL, high-density lipoprotein; LDL, low-density lipoprotein; LV, left ventricular; LVEDVi, LV end-diastolic volume index; LVEF, LV ejection fraction; LVESVi, LV end-systolic volume index; LVMI, LV mass index; LVSD, LV systolic dysfunction; LVSWi, LV stroke work index; MI, myocardial infarction; PCI, percutaneous coronary intervention; RWT, relative wall thickness; SV, stroke volume. Data are expressed as mean ± SD or median (interquartile range), and categorical variables as numbers and percentages.

**Figure 2:**
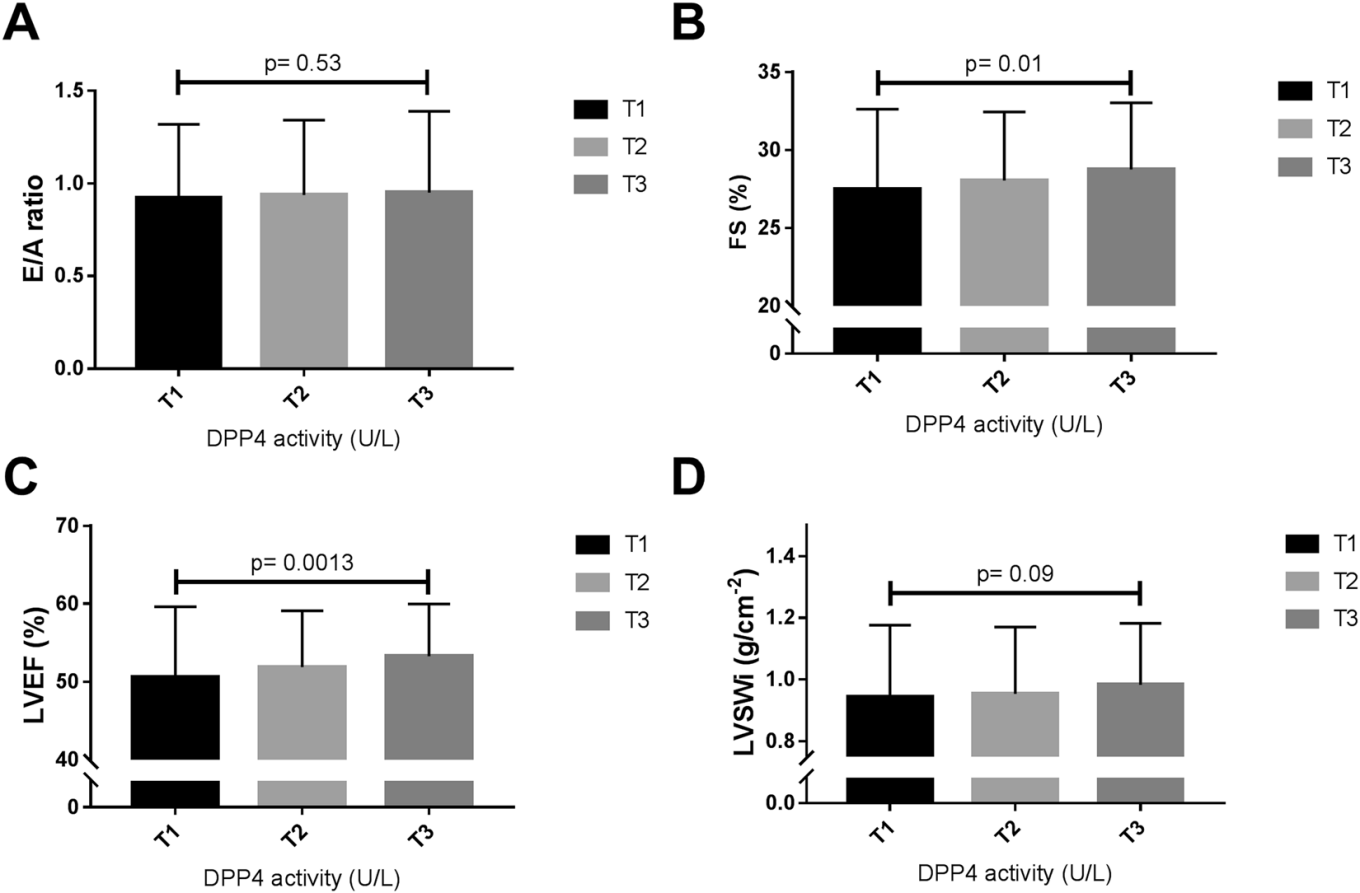
Distribution of echocardiographic parameters assessing left ventricular diastolic and systolic function of STEMI patients according to plasma DPP4 activity tertiles. Bar graphs show the mean ± SD for E/A ratio (**A**), subendocardial fractional shortening (FS) (**B**), left ventricular (LV) ejection fraction (LVEF) (**C**) and LV stroke work corrected by LV end-diastolic volume (LVEDV) (panel D). E, maximum early transmitral velocity in diastole; A, maximum late transmitral velocity in diastole.

Figure 2 shows the echocardiographic parameters assessed in STEMI patients according to DPP4 tertiles. STEMI patients in the lowest tertile had lower LV systolic function in comparison to those in the upper tertile (T1 vs. T3: LVEF: 50.13 ± 9.12 vs. 52.85 ± 6.82%, *p* = 0.001; LV stroke work index (LVSWi): 0.93 ± 0.23 vs. 0.97 ± 0.20 g/cm^−2^, *p* = 0.09; fractional shortening (FS): 27.37 ± 5.18 vs. 28.68 ± 4.25%, *p* = 0.02). The prevalence of LV diastolic function (by E/A ratio) was not significantly different between the three tertiles.

Partial correlation was analyzed between DPP4a and echocardiographic parameters in STEMI patients. After adjusting for potential confounding factors (age, sex, GGT, creatinine, CK-MB, peak NT-proBNP, hypertension, diabetes, and hyperlipidemia), plasma DPP4a was positively correlated with the LVEF (*r* = 0.175, *p* < 0.001) and LVSWi (*r* = 0.161, *p* < 0.001), and inversely correlated with LVEDV (*r* = −0.094, *p* = 0.037), LVESV (*r* = −0.154, *p* = 0.001), LVEDVi (*r* = −0.104, *p* = 0.022), LVESVi (*r* = −0.154, *p* = 0.001), LVM (*r* = −0.094, *p* = 0.021) and LVMI (*r* = −0.105, *p* = 0.021). The partial correlation analysis evaluating the association between plasma DPP4a and the E/A ratio rendered a non-significant *r* coefficient (Table 2).

**Table 2:** Partial correlation coefficients between DPP4 activities and echocardiographic parameters in STEMI patients.

	Partial *r*	*p* value
LVEDV	−0.094	0.037
LVESV	−0.154	0.001
LVEDV_i_	−0.104	0.022
LVESV_i_	−0.154	0.001
LVM	−0.094	0.038
LVMI	−0.105	0.021
RWT	0.032	0.478
SV	0.031	0.488
SW	0.054	0.235
E/A	−0.031	0.505
LVEF	0.175	<0.001
LVSWi	0.161	<0.001
FS	0.118	0.009

After adjustment for age, sex, gamma-glutamyltransferase, creatinine, MB isoenzyme of creatine kinase, pro- brain natriuretic peptide, hypertension, diabetes and hyperlipemia.

DPP4a, plasma dipeptidyl peptidase-4 activity; LV, left ventricular; LVEDV, LV end-diastolic volume; LVESV, LV end-systolic volume; LVEDVi, LVEDV index; LVESVi, LVESV index; LVM, LV mass; LVMI, LVM index; RWT, relative wall thickness; SV, stroke volume; SW, stroke work; E, maximum early transmitral velocity in diastole; A, maximum late transmitral velocity in diastole; LVEF, LV ejection fraction; LVSWi, LV stroke work index; FS, subendocardial fractional shortening.

In multivariate logistic-regression analyses, STEMI patients in the lowest tertile had a fully adjusted odds ratio for LVSD of 8.70 (95% CI: 3.15–24.05, *p* < 0.01) compared with those in the third tertile (32 of 194 [16.5%] in T1 vs. 7 of 194 [3.6%] in T3) (Table 3).

**Table 3:** Association between DPP4a and left ventricular systolic dysfunction in STEMI patients.

	Unadjusted	*p* Value	Model 1*	*p* Value	Model 2^†^	*p* value
	OR (95% CI)		OR (95% CI)		OR (95% CI)	
Left ventricular systolic dysfunction						
T1	5.28 (2.27–12.28)	<0.01	5.31 (2.28–12.40)	<0.01	8.70 (3.15–24.05)	<0.01
T2	3.21 (1.33–7.73)	0.01	3.23 (1.34–7.79)	0.01	4.87 (1.76–13.49)	<0.01
T3	1.00 (reference)		1.00 (reference)		1.00 (reference)	
per 1 U/L	0.93 (0.90–0.96)	<0.01	0.93 (0.90–0.96)	<0.01	0.90 (0.87–0.94)	<0.01

^*^Adjusted for age and sex.

^†^Adjusted for age, sex, body mass index, creatinine, gamma-glutamyltransferase, creatinine, MB isoenzyme of creatine kinase, pro- brain natriuretic peptide, hypertension, hyperlipemia, type 2 diabetes, smoking and previous myocardial infarction.

## Discussion

In the present study, we found that elevated DPP4a levels in STEMI patients were associated with lower peak NT-proBNP levels, with better LV systolic function and lower rates of LVSD independent of confounding factors.

Although plasma DPP4a levels have been directly associated with higher age in healthy volunteers and diabetic patients^12, 13^, inverse associations were found between DPP4a and age in this study. This may be caused by sialylation (a type of glycosylation) of soluble DPP4, which is strongly enhanced in the elderly. The fact that certain types of hypersialylation can inhibit DPP4a is consistent with the fact that plasma DPP4 activity decreases with age^14^. It has been reported that in healthy individuals, DPP4a is correlated with BMI^15, 16^, but that in diabetics no association with BMI occurs^17^. Similar to findings in previous study in diabetics, we found no association of DPP4a with BMI in STEMI patients.

Higher peak NT-proBNP levels occurred in STEMI patients with lower DPP4a. This also occurred in patients with NSTEMI or unstable angina (data not shown). BNP and NT-proBNP are DPP4 substrates, such that BNP (1–32) and NT-proBNP (1–76) could be cleaved by endogenous DPP4 *in vivo* to BNP (3–32) and NT-proBNP (3–76), respectively, resulting in reduced bioactivity^18^. However, commercially available clinical immunoassays do not distinguish between the intact and cleaved forms of NT-proBNP and can only measure the total NT-proBNP concentration. However, any slight change in the relative proportion of NT-proBNP (1–76) and NT-proBNP (3–76) may result in modifications of immune-reactive total NT-proBNP levels^19^. Interestingly, it has been shown that the gliptins (DPP4 inhibitors) that reduces plasma DPP4a results in decreasing of plasma NT-proBNP. In the SAVOR-TIMI 53 study, saxagliptin usage was associated with decreased NT-proBNP levels (4 vs. 10 pg/mL in the placebo group; *p* = 0.001)^20^. In the VIVIDD Trial, diabetic patients taking vildagliptin had lower BNP levels (28 vs. 14% in the placebo group) relative to baseline at 52 weeks^21^. On the other hand, acute treatment with linagliptin does not alter the plasma concentration of BNP and NT-proBNP. The explanation for this may be that observed changes in NT-proBNP over the long term in large randomized controlled trials (RCTs) reflect the natural history of HF and not the effects of gliptins^19^. The association between the plasma DPP4a and NT-proBNP found in STEMI patients here may not be caused by direct cleaving of NT-proBNP by DPP4 but for other reasons, based on acute effects of gliptins on NT-proBNP.

LVSD and HF are common in MI patients^22^. PCI treatment paradoxically results in an increased number of patients at risk of LVSD and HF, with resultant poorer prognoses during short- and long-term follow-up^23^. It has been reported that in diastolic HF patients, DPP4a is positively associated with LV dysfunction^24^. In another study on HF patients, DPP4a was negatively associated with LVEF^10^. In diabetic patients, DPP4a is negatively correlated with LVEF and LVSWi, and is positively associated with subclinical LV systolic and diastolic dysfunction^17^. Unlike these studies, our study showed that DPP4a is positively correlated with LVEF and FS, and is negatively associated with LVSD in STEMI patients. One of the differences between this and previous studies was the inclusion of patients. In the first study, HF patients with coronary artery disease were excluded. In the second study, only 29% of the HF was caused by ischemic heart disease. In the third study, diabetic patients with cardiovascular disease were excluded. The patients in the present study were post-MI patients with coronary artery disease. As DPP4a is down-regulated after MI^11^, it is likely that the degree of DPP4a reduction is associated with the severity of LVSD after STEMI, and thus lower DPP4a reflects worse cardiac function.

It has been debated whether DPP4 inhibitor usage, which reduces plasma DPP4a, may cause HF. In the SAVOR-TIMI 53 study, saxagliptin (a DPP4 inhibitor) lead to a 27% increase of hospitalization for HF (*p* = 0.009) in diabetic patients who had a history of, or were at risk for, cardiovascular events^20^. In the EXAMINE trial, alogliptin (a DPP4 inhibitor) use was associated with increased admissions for HF in T2DM patients with recent acute coronary syndromes, but the differences were not significant: HR 1.19 (95% CI: 0.89–1.58), *p* = 0.24. Interestingly, in the subgroup analysis of patients without a history of HF, alogliptin use was associated with higher rates of HF admissions: HR 1.76 (95% CI: 1.07–2.90), *p* for interaction 0.07^25^. However, in another large RCT, the TECOS study, sitagliptin did not increase the rate of hospitalization for HF in T2DM patients with established cardiovascular disease: HR 1.00 (95% CI: 0.83–1.20)^26^. In a small RCT, the VIVIDD Trial, vildagliptin usage in T2DM patients with established HF did not worsen HF^21^. A recent meta-analysis showed that there is only weak evidence for an increased risk of HF with gliptin usage^27^. Given the cross-sectional design, our data cannot rule out a predictive role of DPP4a on HF. Future studies will be needed to explore the usefulness of DPP4a as a diagnostic biomarker for HF in patients with STEMI.

The DPP4 may affect LV function through glucagon-like peptide 1 (GLP-1). DPP4 inhibitor pretreatment reduces myocardial injury and improves cardiac function in I/R rats through activation of PI3K/Akt signaling pathway by GLP-1/GLP-1 receptor^28^. GLP-1 lowers concentrations of glucose by stimulating secretion of insulin and inhibiting release of glucagon, and once in the circulation, is degraded within 2 to 3 min by DPP4^29^. GLP-1 analogues improve cardiac function after ischemia–reperfusion injury in the rat^30^. Our recent studies show that GLP-1 analog usage during the hospital stay can improve heart function in STEMI patients^31, 32^. However, DPP4 acts not only on glucagon (GLP-1/GLP-2, GIP, glucagon) but also on several other substrates that are involved in the regulation of endothelial and cardiac function, angiogenesis, inflammatory pathways, and metabolism. This may be detrimental. The relative contributions of many of these non-incretin DPP4 substrates to the cardiovascular effects of DPP4 remain to be elucidated^33^. They may contribute to the association between DPP4a and LVSD after acute MI in humans. One the other hand, DPP4a is elevated in HF^10^ and diabetes^17^, but decreased after MI^11^. Given that the reduction of DPP4a after MI may mainly shed from capillary and microvascular endothelial cells, is associatied with coronary microvascular obstruction^34, 35^, and that myocardial microvascular obstruction is associated with LVSD^36, 37^, DPP4a may have an opposite relationship with LV function in patients with MI compared with HF and diabetes.

## Methods

### Subjects

PLA general hospital (PLAGH) is a large national center for tertiary care in Beijing, China. A total of 665 consecutive patients hospitalized at PLAGH between July 2014 and October 2015 with a diagnosis of STEMI and needing PCI were considered for inclusion in this study. STEMI was defined by typical chest pain >30 min, elevated troponin T, with ST-segment elevation of more than 0.1 mm in at least two contiguous electrocardiographic leads. Venous blood samples were collected at admission, and frozen at −80 °C. We excluded patients with malignancies (*n* = 20), those whose blood was not collected (*n* = 26), and those who were being treated with GLP-1 analogues (*n* = 14) or DPP4 inhibitors (*n* = 21). A total of 584 STEMI patients were included. Our study protocol was approved by the ethics committee of PLAGH and the Beijing ethics association and the study was found to be in accordance with the Helsinki Declaration. Written informed consent was obtained from all subjects. The PCI procedures were performed according to the current standard guidelines^38^.

### Echocardiographic study

Complete clinical values, echocardiographic characteristics and laboratory biochemistry indices were collected from each patient. Two-dimensional echocardiographic imaging, targeted M-mode recordings, and Doppler ultrasound measurements were obtained in each patient within the first 3 days after PCI. LV end-diastolic and systolic volumes (LVEDV and LVESV, respectively), posterior wall thickness (PWT), interventricular septum thickness (IVST), LV internal diameter (LVID), and LVEF and endocardial FS were calculated as previously reported^39^. The LV mass (LVM) was calculated in accordance with the Penn convention^40^.

$${\rm{LVM}}=1.04\times ({[{\rm{IVST}}+{\rm{PWT}}+{\rm{LVID}}]}^{3}-{{\rm{LVID}}}^{3})-\mathrm{13.6.}$$

LVEDV, LVESV, and LVM were indexed by body surface area (BSA) (LVEDVi, LVESVi, and LVMI, respectively). Relative wall thickness (RWT) was calculated as PWT/LV internal radius. The following pulsed Doppler measurements of the mitral flow were obtained: maximum early transmitral flow velocity in diastole (E) and maximum late transmitral velocity flow in diastole (A). Stroke volume was calculated as the difference between LVEDV and LVESV. Stroke work (SW) was calculated (as systolic blood pressure multiplied by stroke volume) and was converted into gram-meters (g-m) by multiplying by 0.0144. As a measure of contractility, LV stroke index (LVSWi) was calculated as LV stroke work divided by LVEDV, as previously described^41^. A LVEF fraction of 40 percent or less is considered LVSD^42^:

### Biochemical measurements

Plasma DPP4 activity was measured by detecting p-nitroaniline (pNa) resolved from GP-pNa (Bachem L1880, Swiss), a DPP4 substrate^43^. In brief, 5 µl blood samples were added to 150 µl of 50 mM tris-HCL containing 1 mM GP-pNa, and then the spectrophotometer at 405 nm was read immediately and 1 hour later. The activity is expressed as U/L, indicating that 1 mmol pNa is produced per minute at 37 °C. Biochemical measurements, including those of plasma glucose (GLUC3 Assay, Roche, Germany), cholesterol (CREP2/CHOL2/TRIGL/LDL_C/HDLC3 Assay, Roche, Germany), the MB isoenzyme of creatine kinase (CK-MB) (Elecsys CK-MB Assay, Roche, Germany), cardiac troponin T (cTNT) (Elecsys Troponin T hs Assay, Roche, Germany), NT-proBNP (Elecsys proBNP II Assay, Roche, Germany) and creatinine (CREP2 Assay, Roche, Germany) were obtained using standard clinical analytical methods (Cobas Roche, Germany). For plasma glucose, creatinine and lipid profile measurements we used the Cobas c701 platform (Roche, Swiss). For myocardial injury markers (CK-MB, cTnT and NT-proBNP) measurements we used the Cobas e602 platform (Roche, Swiss). The normal range of the NT-proBNP test was <150 pg/mL.

### Statistical analysis

Normal distribution was tested by plotting both PP and QQ plots. Continuous data were expressed as mean ± standard deviation or, if non-normally distributed, as median (with interquartile range). We divided the study individuals of each group into three categories according to DPP4a tertiles and used these categories in the following analyses. Differences among groups were tested by ANOVA for normally distributed data; otherwise, a nonparametric Kruskal–Wallis test was used for continuous data and a chi-square test for dichotomous data. The statistical analysis was done using SPSS software (13.0 version; SPSS Inc., Chicago, IL, USA). A two-sided *p*-value < 0.05 was considered as significantly different.
